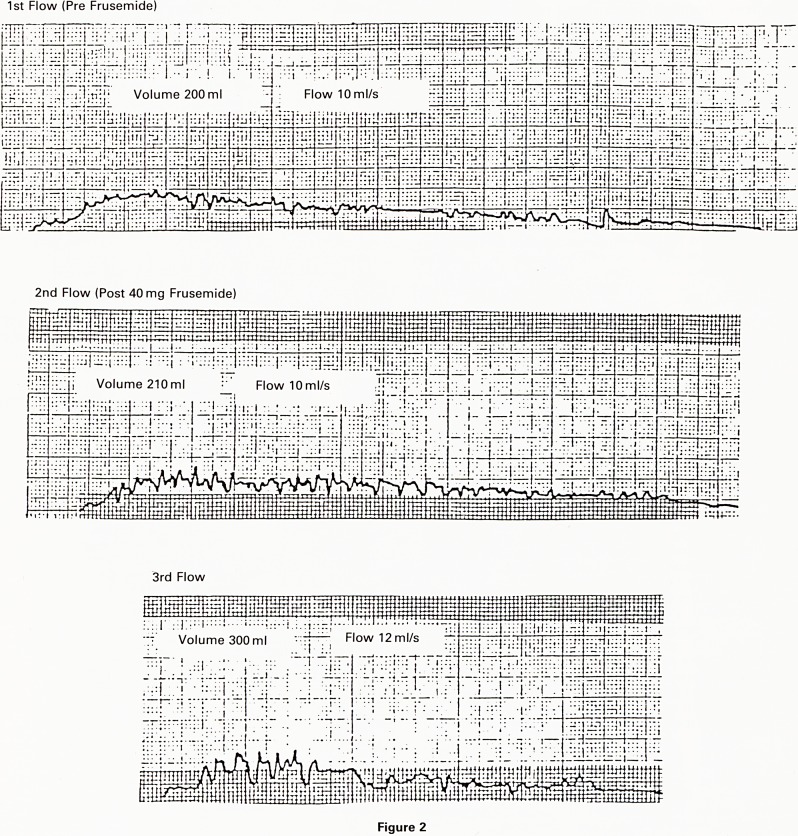# A Urine Flow Clinic

**Published:** 1988-02

**Authors:** M. E. Lucarotti, R. C. L. Feneley, P. Abrams

**Affiliations:** Department of Urology, Southmead Hospital, Bristol

## Abstract

The assessment of male patients with lower urinary tract symptoms presents a common urological request and prostatic surgery places heavy demands on Health Service resources. Sixty male patients attended a urine flow clinic during their preliminary clinical assessment, to identify those with objective evidence of bladder outflow obstruction. Nineteen were shown to have a reduced urine flow rate and of these 13 proceeded to prostatectomy. The clinic has a useful role as a screening investigation.


### A Urine Flow Clinic

**M. E. Lucarotti FRCS*, R. C. L. Feneley M.Chir, P. Abrams MD.**
Department of Urology, Southmead Hospital, Bristol.

### ABSTRACT

The assessment of male patients with lower urinary tract symptoms presents a common urological request and prostatic surgery places heavy demands on Health Service resources. Sixty male patients attended a urine flow clinic during their preliminary clinical assessment, to identify those with objective evidence of bladder outflow obstruction. Nineteen were shown to have a reduced urine flow rate and of these 13 proceeded to prostatectomy. The clinic has a useful role as a screening investigation.

### INTRODUCTION

Patients are referred to a urological clinic for further investigation of a symptom complex, which includes frequency and urgency of micturition, nocturia, hesitancy and a poor stream. These symptoms raise suspicion of prostatic obstruction and are often referred to as 'prostatism'.

Symptoms are notoriously unreliable and give a poor indication of diagnosis in patients with lower urinary tract dysfunction. Objective evaluation with urodynamic studies showed that hesitancy and a poor stream were the only symptoms significantly associated with proven bladder outflow obstruction (1). Another feature often mentioned in the referral letter is the finding of an enlarged prostate. The size of a prostate is poorly related to the severity of bladder outflow obstruction (2).

The measurement of the urine flow rate provides a simple, non-invasive screening investigation. The flow rate is dependent on both the age of the patient and the volume voided. If the volume is less than 150 ml., the result needs to be interpreted with caution. The urine flow clinic can be administered by the nursing staff, following adequate instruction.

### PATIENTS AND METHODS

Sixty male patients were referred for urological assessment. These patients were asked to attend the urine flow clinic and to arrive with a reasonably full bladder. They had been previously warned that they might be required to stay at the hospital for two-to-three hours, in order to perform multiple flow measurements.

On arrival at the clinic, an initial flow rate is measured. The flow meter is housed in the privacy of a normal toilet, with the recording apparatus in an adjacent room, to avoid distracting the patient. Particular effort is made to ensure that the patient's performance is typical of his normal habit. Patients are asked to complete a frequency and volume chart during their initial assessment and this provides a record of the functional bladder capacity.

The patient is then given a tablet of Frusemide (40 mg.) and encouraged to drink up to 2 litres of water. Following this, the patient can normally perform two or three further urine flow measurements.

A single flow rate can be misleading and the advantage of assessing multiple flow measurements has been recognised. In a series of 27 male patients, with suspected bladder outflow obstruction, Powell and Ball (3) showed that 20 had objective evidence of bladder outflow obstruction on pressure/flow analysis. A single record of the urine flow rate predicted the pressure/flow results correctly in only 8 cases (30%), whereas multiple recordings gave a correct prediction in 25 cases (93%). The administration of Frusemide does tend to increase the volume of urine voided and thus provides more conclusive results. Furthermore, the nursing staff have found that it is beneficial to invite 3 or 4 patients to attend the clinic at the same time, because this introduces a competitive element to the performance!

Several different types of urine flow meter are now available, using different principles. Reliability of the

**Figure 1**

Correspondence to: Mr. R. C. L. Feneley, Department of Urology, Southmead Hospital, Bristol, BS10 5NB.
*Present address: Registrar in General Surgery, East Birmingham Hospital, Bordesley Green East, Birmingham.

equipment and accuracy of measurement are important factors. Automatic activation of the chart recorder, prior to or by the urine flow, is an advantage by making the patient less aware of the machinery. The result of the flow tracing should be interpreted by noting the maximum flow rate and the length of the flow time. A normal tracing shows that maximum flow is reached within 5–10 seconds of the onset of micturition (Fig. 1). The flow time may be prolonged if the urinary stream is interrupted. In this study the provisional diagnosis of outflow tract obstruction was made if the maximum urine flow rate was below 13 ml/s (Fig. 2).

### RESULTS

Sixty male patients were assessed, with a mean age of 65 years, (Table I). They presented with one or more symptoms, which included frequency, urgency, nocturia, hesitancy and a poor stream (Table II). Thirty patients (50%) were reported clinically to have an enlarged prostate. Measurement of the urine flow rate showed that only 19 of the 60 patients referred had flow rates of 12 ml/s or less (Table III). The reduced urine flow rate of these 19 patients was accepted as confirmatory evidence of bladder outflow obstruction. Thirteen patients subsequently

**Figure 2**

**Table 1**

| | |
|---|---|
| Total no. of patients | 60 |
| Range of ages | 31–84 years |
| Mean age | 65 years |
| Age 55 years or over | 49 (82%) |

**Table 2**

**Presentation**

| | Symptoms | No. of patients | |
|---|---|---|---|
| Irritative | ( frequency | 36 | (60%) |
| | ( urgency | 8 | (13%) |
| | ( nocturia | 6 | (10%) |
| Obstructive | ( hesitancy | 22 | (37%) |
| | ( poor stream | 16 | (27%) |

**Table 3**

**Results**

| Maximum urine flow rates | No. of patients | |
|---|---|---|
| 13 ml/s or more | 41 | (68%) |
| 12 ml/s or less | 19 | (32%) |

**Table 4**

**Management of patients with reduced urine flow rates**

| | |
|---|---|
| No. of patients | 19 |
| Prostatectomy | 13 |
| Dilatation of urethral stricture | 2 |
| Spontaneous improvement | 4 |

proceeded to prostatectomy, 2 were found to have urethral strictures and 4 symptomatically improved spontaneously, (Table IV).

### DISCUSSION

Due to the rise in the age of the population and public awareness, an increasing number of patients are being referred with lower urinary tract symptoms. Thus, there is a clinical need for screening investigations for these patients and the urine flow meter provides a simple objective evaluation of voiding dysfunction. Many patients have been told that they may have a prostatic problem before the referral, on the basis of a palpably enlarged gland. The term 'prostatism' infers an aetiology of prostatic obstruction and should be avoided, because the assumption is unjustified until investigations have been completed. Modest experience in the use of a urine flow meter produces results of clinical value. The urine flow rate cannot normally be measured at the time of the outpatient consultation, because apprehension reduces the chance of a representative recording of the individual's normal flow rate and the patient is normally asked to provide a urine specimen for routine tests. To overcome these objections, patients are referred to a specific clinic and they are given a careful explanation of the procedure. The measurement of the urine flow rate does assist the diagnosis of bladder outflow obstruction. Elderly patients do develop a habitual pattern of frequent micturition, which may or may not be related to outflow tract obstruction. A proportion of the patients become fearful of developing acute retention of urine, particularly if an acquaintance has experienced that condition.

Ball et al (4) reviewed 107 patients with symptoms of prostatic obstruction, in whom prostatectomy was not considered to be clinically indicated, after an interval of 5 years from their initial assessment. Two patients had developed an acute retention and a further 8 had required surgery. In the majority, symptoms did not deteriorate, but the flow rate measurement did identify those patients at risk of clinical deterioration.

The assessment of patients with suspected outflow tract obstruction cannot rely on any single result in isolation. The routine procedure includes a comprehensive clinical examination, urinalysis, microbiological examination of a mid-stream specimen of urine, full blood picture, blood urea and a plain film of the urinary tract. Patients with evidence of chronic retention of urine and a palpable distended bladder, uraemia or a carcinoma of the prostate, were excluded from this study. The plain x-ray of the urinary tract after micturition can reveal the soft tissue shadow of the bladder size and ultrasound (5) is a reliable, non-invasive method of assessing bladder wall thickness and residual urine. Excretion urography is of limited value in the assessment of bladder outflow obstruction (6).

The establishment of a urine flow clinic to obtain more than one measurement of the urinary stream has produced information of clinical value. Von Garrelts (7) recognised the inaccuracy of the patient's own estimate of their performance. Urodynamic investigations have introduced objective criteria in the selection of patients who are likely to benefit from lower urinary tract surgery and the measurement of the urine flow rate has been identified as the simplest screening method in male patients with suspect obstructed micturition.

The introduction of a urine flow clinic has been shown to be of value as a screening procedure for male patients with bladder outflow obstruction.

### REFERENCES

1. ABRAMS, P. H. and FENELEY, R. C. L. (1978) The significance of symptoms associated with bladder outflow obstruction. *Urol.Int.* **33**, 171–174.
2. TURNER WARWICK, R., WHITESIDE, C. G., ARNOLD, E. P. *et al* A urodynamic view of prostatic obstruction and the results of prostatectomy. *Br.J.Urol.* **45**, 631–645.
3. POWELL, P. H. and BALL, A. (1983) Urodynamics. Ed P. H. Abrams, R. C. L. Feneley, M. Torrens. Springer-Verlag p. 159.
4. BALL, A. J., FENELEY, R. C. L. and ABRAMS, P. H. (1981) The natural history of untreated "prostatism". *Br.J.Urol.* **53**, 613–616.
5. MATTHEWS, P. N., QUAYLE, J. B., JOSEPH, A. E. A., *et al.* (1982) The use of ultrasound in the investigation of prostatism. *Br.J.Urol.* **54**, 536–538.
6. ABRAMS, P. H., ROYLANCE, J. and FENELEY, R. C. L. (1976) Excretion urography in the investigation of prostatism. *Br.J.Urol.* **48**, 681–684.
7. GARRELTS, B. von. (1956) Analysis of micturition. A new method of recording the voiding of the bladder. *Acta.Chir. Scand.* **112**, 326–340.

## Figures and Tables

**Figure 1 f1:**
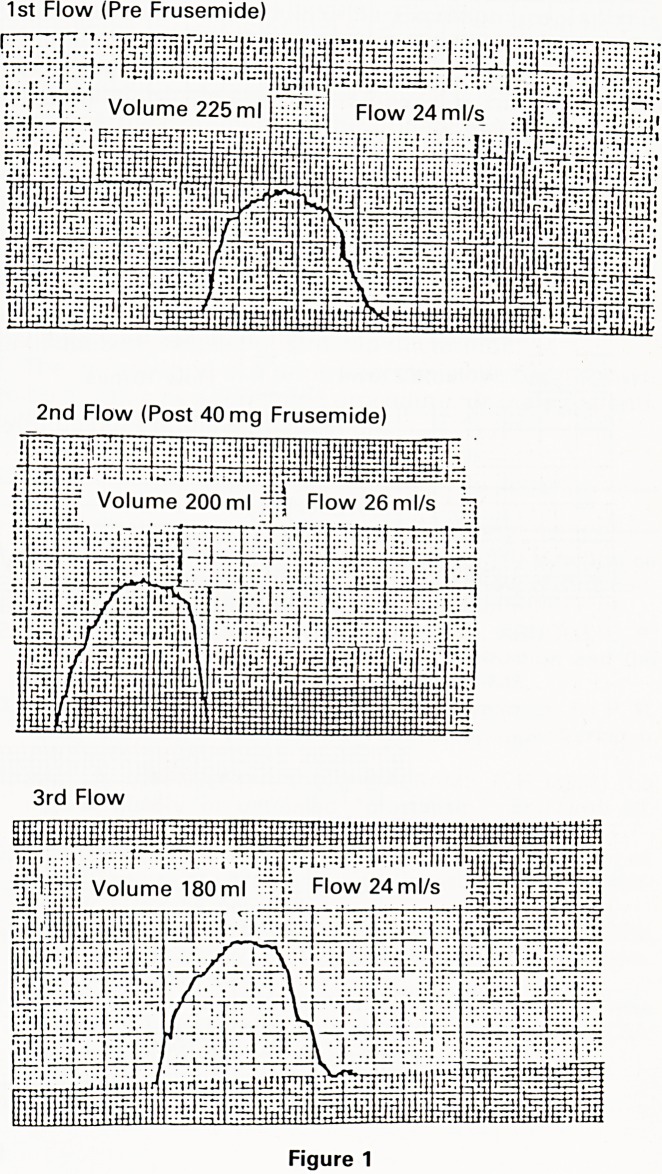


**Figure 2 f2:**